# Regional versus general anesthesia in older patients for hip fracture surgery: a systematic review and meta-analysis of randomized controlled trials

**DOI:** 10.1186/s13018-023-03903-5

**Published:** 2023-06-13

**Authors:** Sheng-Liang Zhou, Shao-Yun Zhang, Hai-Bo Si, Bin Shen

**Affiliations:** 1 Department of Orthopedic Surgery and Orthopedic Research Institute, West China Hospital, Sichuan University, No. 37 Guoxue Road, Sichuan Province 610041 Chengdu, People’s Republic of China; 2 Department of Orthopedics, The Third Hospital of Mianyang, Sichuan Mental Health Center, Mianyang, Sichuan Province People’s Republic of China

**Keywords:** Anesthesia, General, Hip fracture, Meta-analysis, Regional

## Abstract

**Background:**

The optimal anesthesia technique for older patients undergoing hip fracture surgery remains controversial. We performed a systematic review and meta-analysis of updated randomized controlled trials (RCTs) to assess whether regional anesthesia was superior to general anesthesia in hip fracture surgery.

**Methods:**

We searched PubMed, EMBASE, Web of Science, and the Cochrane Central Register of Controlled Trials from January 2000 until April 2022. RCTs directly comparing regional and general anesthesia in hip fracture surgery were included in the analysis. The incidence of delirium and mortality were the primary outcomes and other perioperative outcomes including complications were secondary outcomes.

**Results:**

Thirteen studies involving 3736 patients were included in this study. There was no significant difference in the incidence of delirium (odds ratio [OR] 1.09; 95% confidence interval [CI] 0.86, 1.37) and mortality (OR 1.08; 95% CI 0.71, 1.64) between the two groups. Patients receiving regional anesthesia in hip fracture surgery were associated with a reduction in operative time (weighted mean difference [WMD]: − 4.74; 95% CI − 8.85, − 0.63), intraoperative blood loss (WMD: − 0.25; 95% CI − 0.37, − 0.12), postoperative pain score (WMD: − 1.77; 95% CI − 2.79, − 0.74), length of stay (WMD: − 0.10; 95% CI − 0.18, − 0.02), and risk of acute kidney injury (AKI) (OR 0.56; 95% CI 0.36, 0.87). No significant difference was observed in the other perioperative outcomes.

**Conclusions:**

For older patients undergoing hip fracture surgery, RA did not significantly reduce the incidence of postoperative delirium and mortality compared to GA. Due to the limitations of this study, the evidence on delirium and mortality was still inconclusive and further high-quality studies are needed.

**Supplementary Information:**

The online version contains supplementary material available at 10.1186/s13018-023-03903-5.

## Introduction

It has been estimated that 1.6 million people worldwide sustained a hip fracture in 2000 [1]. The incidence of hip fracture may decline or plateau in some regions; however, with a rapidly aging global population, the number of patients with hip fractures will increase [2–5]. The number is expected to reach 4.5 million worldwide in 2050 and the cost for a hospital stay will be a significant burden on society [6–8]. Almost all hip fracture patients are offered surgical treatment to restore their functional status which requires anesthesia [9]. Regional neuraxial block and general anesthesia (GA) are the most common anesthetic techniques that are applied for hip fracture surgery; however, no consensus has been reached on whether regional or general anesthesia is the optimal technique.

GA has been reported to have a higher risk of postoperative delirium [10], and mortality [11], as well as a longer perioperative length of stay (LOS) [12], and a lower risk of some postoperative complications [13] than regional anesthesia (RA) based on previous observational studies. In recent years, a meta-analysis of randomized controlled trials (RCTs) indicated that there was a significant difference in blood loss between GA and RA and no difference in the incidence of delirium, or 30-day mortality [14]. However, this meta-analysis included small RCTs and compared limited outcomes. The quality of the evidence was rated as low by using the Grading of Recommendations, Assessment, Development, and Evaluations (GRADE) system, which indicates that the results may be changed by performing further high-quality RCTs. The effect of anesthetic techniques that are applied for hip fracture surgery on the incidence of postoperative delirium and mortality is still controversial. Recently, several well-designed RCTs with larger sample sizes comparing the effects of RA with GA for older patients undergoing hip fracture surgery have been published [15–17]. Therefore, we aimed to conduct a systematic review and meta-analysis of RCTs to explore whether RA was inferior to GA for patients with hip fracture surgery.

## Methods

### Protocol and registration

The study protocol has not been previously published. This systematic review and meta-analysis was conducted according to the principles of the Preferred Reporting Items for Systematic Reviews and Meta-Analyses (PRISMA) statement [18]. Our study has been registered in PROSPERO (CRD42022315800). In our study, RA included spinal anesthesia (SA), epidural anesthesia, or combined spinal epidural techniques and the use of sedation was noted.

### Search strategy

We searched PubMed, EMBASE, Web of Science, and the Cochrane Central Register of Controlled Trials in the Cochrane Library and limited the search date from January 2000 until April 2022 to focus on modern anesthetic techniques in recent studies. The following search terms were used as subject headings and key words: “Hip fracture,” “General anesthesia,” “Regional anesthesia” OR “spinal anesthesia” OR “epidural anesthesia.” The search was restricted to human studies in the English language. Additional studies were retrieved by screening the references of all of the eligible studies and review articles.

### Inclusion and exclusion criteria

S-LZ and S-YZ screened the titles and abstracts of the search findings and full texts were reviewed for all eligible studies according to predefined study selection criteria. Studies were included if they clearly documented the comparison between GA and RA for older patients undergoing hip fracture surgery and reported on one of the outcomes described as follows. Only RCTs were included and observational studies, abstracts, reviews, and case reports were excluded. Disagreements about the eligibility of the studies were resolved by the third investigator.

### Outcomes and definitions

The primary outcomes were the incidence of postoperative delirium (any criteria as defined by the study authors) and postoperative mortality. Secondary outcomes included intraoperative outcomes and postoperative outcomes. The intraoperative outcomes were operative time, intraoperative hypotension, duration of anesthesia, blood loss, and blood transfusion. Postoperative outcomes included postoperative pain score, LOS, and postoperative adverse events. Postoperative adverse events included postoperative nausea and vomiting (PONV), deep vein thrombosis (DVT), pneumonia, acute myocardial infarction, heart failure, stroke, acute kidney injury (AKI), and surgical-site infection.

### Data extraction and quality assessment

S-LZ and S-YZ extracted data from the eligible studies and these data included study design, patient characteristics, American Society of Anesthesiologists (ASA) physical status I–IV (I [healthy], II [mild systemic disease], III [severe systemic disease], and IV [severe systemic disease that is a constant threat to life]), type of fracture, anesthesia type, and predefined outcomes.

The same two investigators assessed the methodological quality of the RCTs by using the Cochrane Collaboration risk of bias tool [19]. “A total of seven domains (random sequence generation, allocation concealment, blinding of participants and personnel, blinding of outcome assessment, incomplete outcome data, selective reporting, and other bias) were classified as demonstrating high, unclear, or low risk.” Any disagreement about data extraction and quality assessment was resolved via discussion with the third investigator.

### Statistical analysis

Review Manager software, version 5.4.1 (The Cochrane Collaboration, Oxford, UK) and STATA/MP, version 17.0 were used to perform this meta-analysis. Continuous variables were calculated with weighted mean differences (WMDs) of the mean values and standard deviations (SDs); in addition, odds ratios (ORs) with corresponding 95% confidence intervals (CIs) were calculated for dichotomous outcomes. The results reported with medians and interquartile ranges (IQRs) were converted to mean and SD by using the previously described method [20]. For continuous outcomes with a skewed nature, the means and SDs were transformed to the log scale according to the well-established equations [21]. The results of the studies were pooled only if at least two studies reported on the same outcome. Heterogeneity was assessed by using the Chi-square test, with a *P* value of < 0.1 indicating statistical significance; moreover, the *I*^2^ statistic was estimated for the extent of heterogeneity. A fixed effects model was used when there was no statistical heterogeneity among the studies (*P* > 0.1, *I*^2 ^< 50%), and a random effects model was used when statistical heterogeneity existed (*P* < 0.1, *I*^2^ > 50%). Sensitivity analyses were conducted by using the leave-one-out method. Studies comparing SA as the only anesthetic technique with GA were included in the subgroup analyses. The GRADE system was used to assess the overall quality of the evidence for each outcome. Publication bias was assessed via the funnel plots and by using the Egger test [22].

## Results

### Study selection

**Fig. 1:**
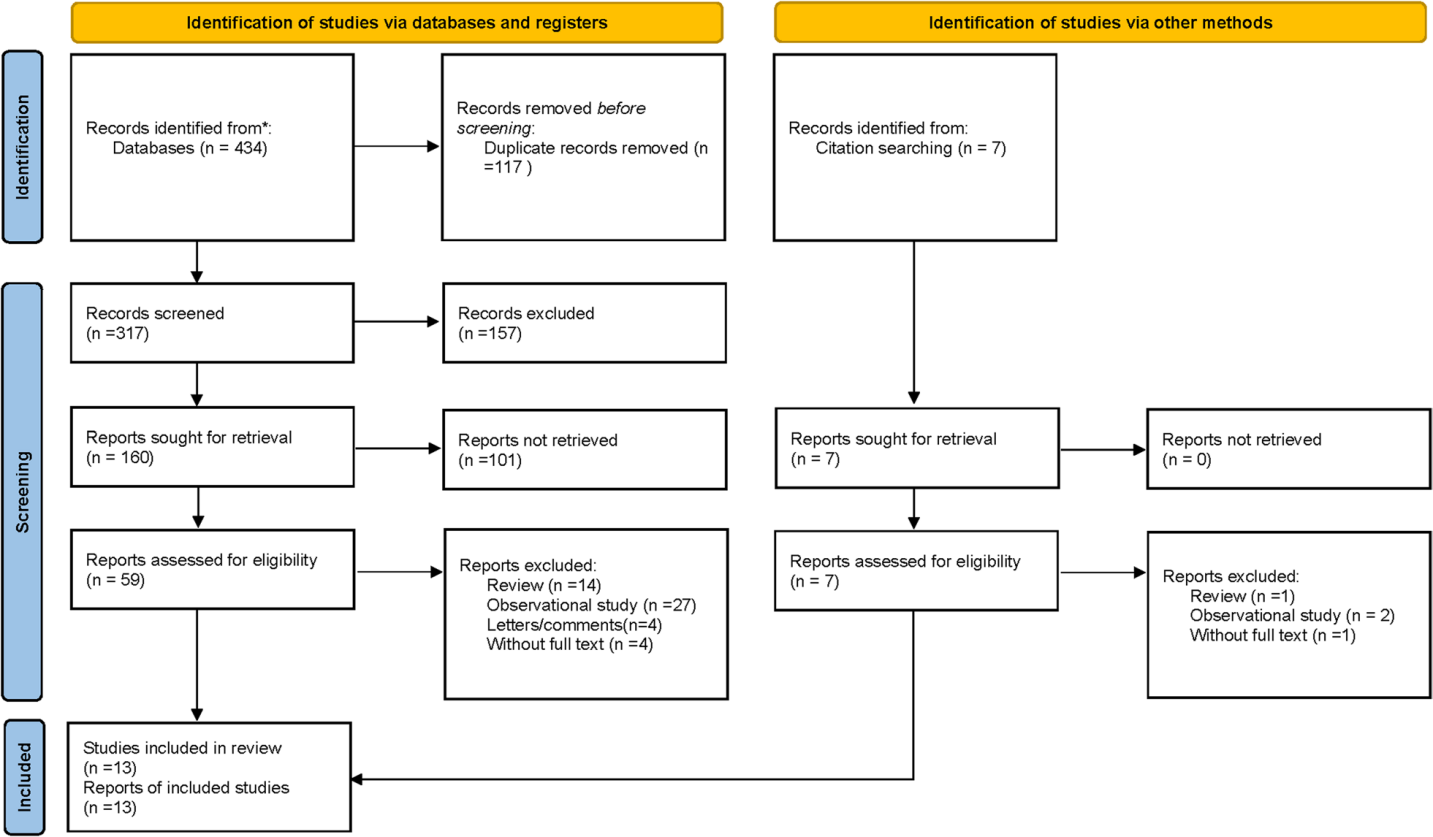
Flow diagram of the literature search and selection process

We identified 441 articles of which 62 full-text studies were assessed for eligibility, and a total of 13 studies were identified for this meta-analysis according to the inclusion criteria (Fig. 1).

### Study characteristics

These studies involved a total of 3736 patients: 1885 individuals in the GA group and 1851 patients in the RA group. Eleven of thirteen studies [16, 17, 23–31] applied SA as the sole anesthesia type of RA, two [17, 26] of which provided SA with sedation. Another two studies [15, 32] used SA, epidural anesthesia, or combined spinal epidural techniques as RA. Six studies [15, 17, 26, 27, 30, 32] clearly reported the types of hip fractures, including femoral neck, femoral head, intertrochanteric, and subtrochanteric fractures. The types of surgery included fixation, total hip arthroplasty, and hemiarthroplasty. The general characteristics of these studies are summarized in Table 1.

**Table 1:** Characteristics of included studies

Study	Country	Design	Intervention	*N*	Male/female	Age (years)	ASA	Fracture type	Surgery type	Anesthetic techniques
Li 2022	China	RCT	RA	471	128/343	77.00 (72.00–82.00)	I–IV	1,2,3,4	Fixation	SA, EA, combined S-EA
			GA	471	119/352	77.00 (71.00–82.00)				GA
Neuman 2021	America & Canada	RCT	RA	795	258/537	77.70 ± 10.70	I–IV	1,2,3,4	Fixation, THA, HA	SA with sedation
			GA	805	270/535	78.4 ± 10.6				GA
Tang 2021	China	RCT	RA	55	16/39	78.00 ± 6.45	II–IV	NA	Fixation, THA, HA	SA
			GA	55	20/35	76.60 ± 6.98				GA
Tzimas 2018	Greece	RCT	RA	37	NA	77.11 ± 6.5	I–III	NA	NA	SA
			GA	33		75.09 ± 6.08				GA
Meuret 2018	France	RCT	RA	19	2/17	83.00 ± 6.00	I–III	NA	Fixation, THA,	SA
			GA	21	6/15	85.00 ± 5.00				GA
Haghighi 2017	Iran	RCT	RA	50	42/8	66.22 ± 5.17	I–III	NA	NA	SA
			GA	50	38/12	65.98 ± 4.76				GA
Neuman 2016	America	Pilot RCT	RA	6	4/2	80.50 (62.00–92.00)	NA	1,3,4	NA	SA with sedation
			GA	6	5/1	62.50 (57.00–88.00)				GA
Parker 2015	Britain	RCT	RA	158	30/128	82.90 (52.00–105.00)	I–IV	1,2,3,4	Fixation, THA,	SA
			GA	164	57/107	83.00 (59.00–99.00)				GA
Messina 2013	Italia	RCT	RA	10	3/7	81.80 ± 6.30	III, IV	NA	NA	SA
			GA	10	4/6	83.90 ± 9.40				GA
Biboulet 2012	France	RCT	RA	15	4/11	87.00 ± 7.00	III, IV	NA	Fixation, HA	SA
			GA	28	8/20	85.50 ± 5.90				GA
Heidari 2011	Iran	RCT	RA	190	109/53	NA	NA	1,3,4	NA	SA, EA
			GA	197	148/49					GA
Hoppenstein 2005	Israel	RCT	RA	30	NA	81.50 ± 8.00	I–III	1	NA	SA
			GA	30		83.50 ± 8.00				GA
Casati 2003	Italy	RCT	RA	15	1/14	84.00 (71.00–94.00)	II, III	NA	NA	SA
			GA	15	1/14	84.00 (67.00–88.00)				GA

Statistics are presented as mean ± standard deviation or median (interquartile range) as appropriate

*RCT* Randomized controlled trial, *N* Number of patients, *GA* General anesthesia, *RA* Regional anesthesia, *SA* Spinal anesthesia, *EA* Epidural anesthesia, *combined S-EA* Combined spinal epidural anesthesia, *NA* Not available, *ASA* American Society of Anesthesiologists physical status I to IV (I [healthy], II [mild systemic disease], III [severe systemic disease], IV [severe systemic disease that is a constant threat to life]), *THA* Total hip arthroplasty, *HA* Hemiarthroplasty

1 = femoral neck, 2 = femoral head, 3 = intertrochanteric, 4 = subtrochanteric fracture

### Risk of bias

In terms of selection bias, only one study was at high risk because of randomization with sequential numbers, and three studies were at unclear risk without reporting information about randomization. For allocation concealment, half of the included studies did not report on the method that they used. Additionally, more than half of the studies were assessed to have a high risk of performance and detection bias because they were open-label studies or did not provide related information. The risk of attrition bias was determined to be moderate, and the risk of reporting bias was low. A summary of the risk of bias and explanation are presented in Additional file 1.

### Primary outcomes

**Fig. 2:**
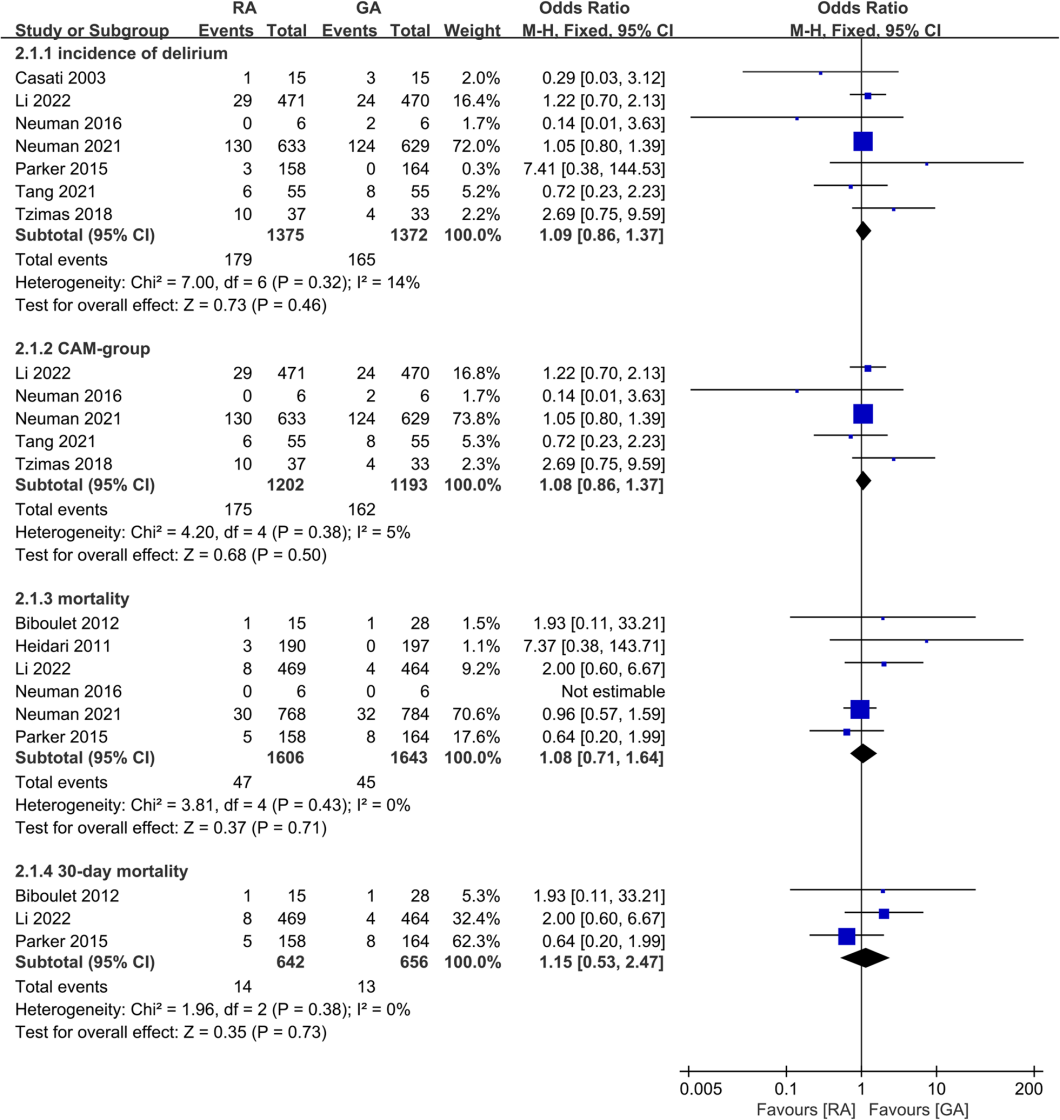
Forest plots displaying pooled effect estimates for primary outcomes. *RA* Regional anesthesia, *GA* General anesthesia

Seven studies reported on the incidence of delirium and the meta-analysis showed that there was no significant difference in this outcome (OR 1.09; 95% CI 0.86, 1.37, *P* = 0.46, *n* = 2747) (Fig. 2). Five studies [15–17, 23, 26] diagnosed delirium by using the Confusion Assessment Method (CAM); in addition, one study [31] diagnosed delirium with the Mini Mental State Examination (MMSE) test, and one study [27] did not provide information on how to define delirium. The meta-analysis of studies with the CAM method did not change the conclusion (OR 1.08; 95% CI 0.86, 1.37, *P* = 0.50, *n* = 2395) (Fig. 2). The GRADE evaluation demonstrated low- and high-quality evidence for the incidence of postoperative delirium and the CAM group, respectively, which is shown in Additional file 2.

There was no significant difference in postoperative mortality between the RA group and GA group in the meta-analysis of six studies [15, 17, 26, 27, 29, 32] (OR 1.08; 95% CI 0.71, 1.64, *P* = 0.71, *n* = 3249) (Fig. 2). Three studies [15, 27, 29] explicitly reported on 30-day mortality, and the meta-analysis did not show a significant difference between patients receiving RA and GA (OR 1.15; 95% CI 0.53, 2.47, *P* = 0.73, *n* = 1289) (Fig. 2). The quality of evidence for postoperative mortality was low and was moderate for 30-day mortality according to the GRADE system (Additional file 2).

### Intraoperative outcomes

**Fig. 3:**
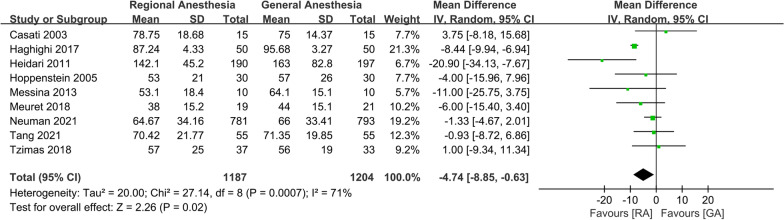
Forest plots displaying pooled effect estimates for operative time. *RA* Regional anesthesia, *GA* General anesthesia

The meta-analysis of 9 studies showed that the operative time of the GA group was significantly longer than that of the RA group (WMD: − 4.74; 95% CI − 8.85, − 0.63, *P* = 0.02, *n* = 2391) (Fig. 3). GRADE evidence for the operative time was very low (downgraded for inconsistency and imprecision) (Additional file 2).

**Fig. 4:**
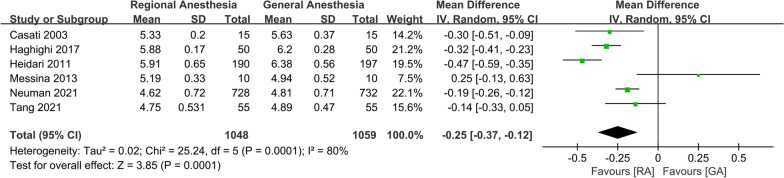
Forest plots displaying pooled effect estimates for intraoperative blood loss. *RA* Regional anesthesia, *GA* General anesthesia

The pooled data of six studies involving 1,048 patients in the RA group and 1059 patients in the GA group demonstrated more intraoperative blood loss in the GA group (WMD: − 0.25; 95% CI − 0.37, − 0.12, *P* = 0.0001, *n* = 2107) (Fig. 4). The quality of evidence for this outcome was low according to the GRADE system (downgraded for high risks of bias and inconsistency) (Additional file 2).

**Fig. 5:**
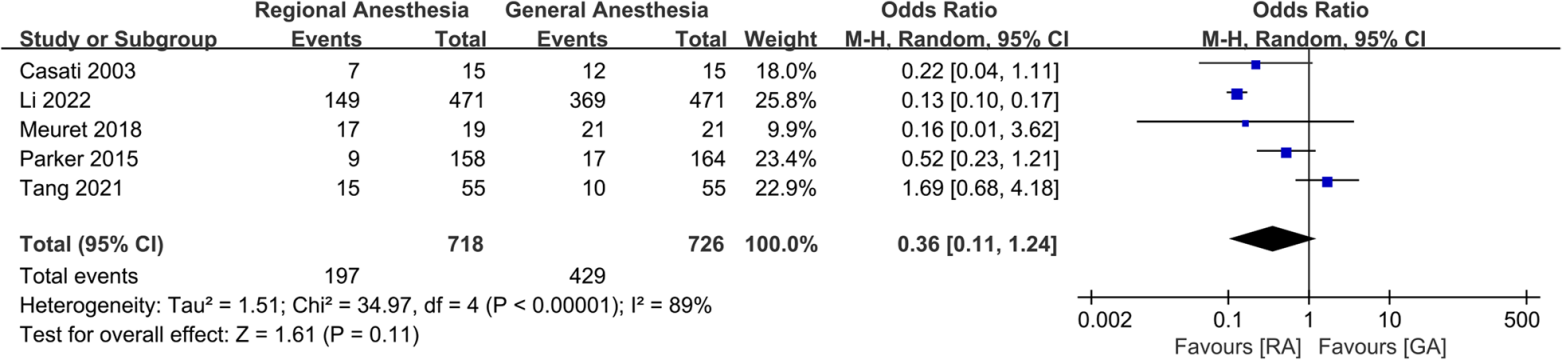
Forest plots displaying pooled effect estimates for incidence of intraoperative hypotension. *RA* Regional anesthesia, *GA* General anesthesia

**Fig. 6:**
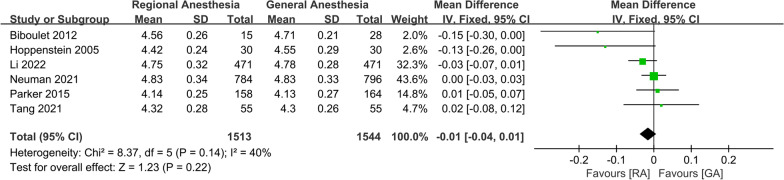
Forest plots displaying pooled effect estimates for duration of anesthesia. *RA* Regional anesthesia, *GA* General anesthesia

**Fig. 7:**
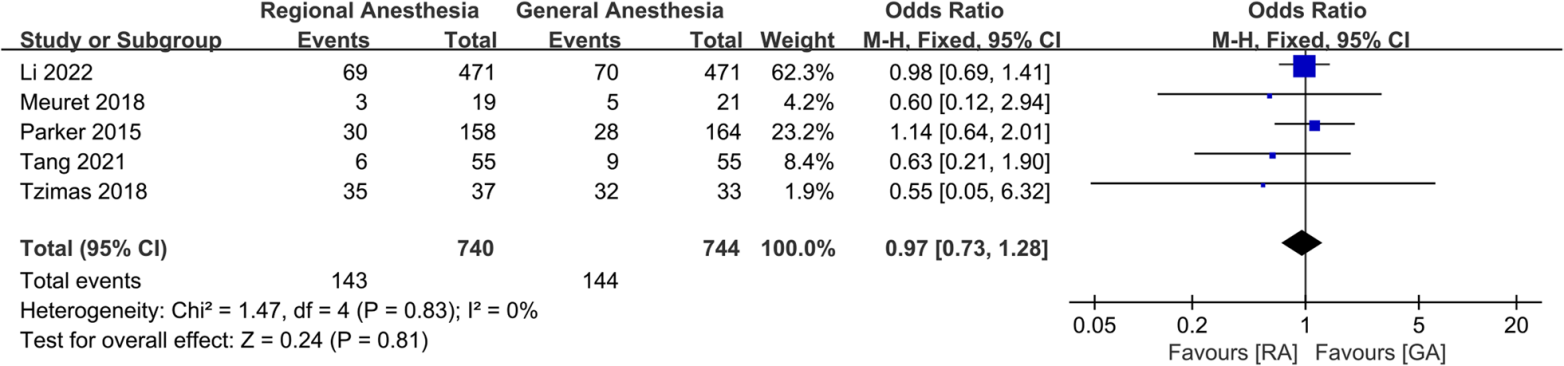
Forest plots displaying pooled effect estimates for intraoperative blood transfusion. *RA* Regional anesthesia, *GA* General anesthesia

No significant difference was observed in the incidence of intraoperative hypotension (OR 0.36; 95% CI 0.11, 1.24, *P* = 0.11, *n* = 1444) (Fig. 5), duration of anesthesia (WMD: − 0.01; 95% CI − 0.04, 0.01, *P* = 0.22, *n* = 2107) (Fig. 6), or intraoperative blood transfusion (OR 0.97; 95% CI 0.73, 1.28, *P* = 0.81, *n* = 1484) (Fig. 7). GRADE evidence for intraoperative hypotension and duration of anesthesia was low and moderate for intraoperative blood transfusion, respectively (Additional file 2).

### Postoperative outcomes

**Fig. 8:**
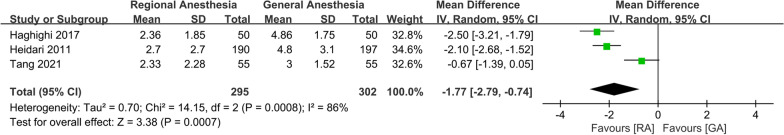
Forest plots displaying pooled effect estimates for postoperative pain score. *RA* Regional anesthesia, *GA* General anesthesia

Four studies reported on the postoperative pain score of patients which was evaluated with a visual analog scale (VAS). Three studies used VAS ranging from 0 (no pain) to 10 (worst pain) and one study ranged from 0 (no pain) to 100(worst pain). In the meta-analysis of three studies with the same range, patients receiving GA had higher postoperative pain scores than those receiving RA (WMD: − 1.77; 95% CI − 2.79, − 0.74, *P* = 0.0007, *n* = 597) (Fig. 8). GRADE evidence was graded as moderate for postoperative pain score (Additional file 2).

**Fig. 9:**
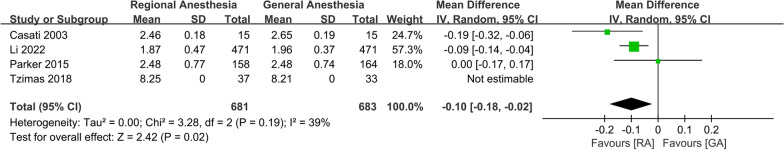
Forest plots displaying pooled effect estimates for length of stay. *RA* Regional anesthesia, *GA* General anesthesia

We included four studies in the meta-analysis of LOS and observed no significant difference between the two groups (WMD: − 0.10; 95% CI − 0.18, − 0.02, *P* = 0.02, *n* = 1364) (Fig. 9). Another two studies also reported the LOS; however, we excluded these studies from the meta-analysis. One study [17] reported on hospital stay according to country, and the other study [32] reported on hospital stay before operation and hospital stay after operation. The quality of evidence for LOS was very low according to the GRADE system (Additional file 2).

**Fig. 10:**
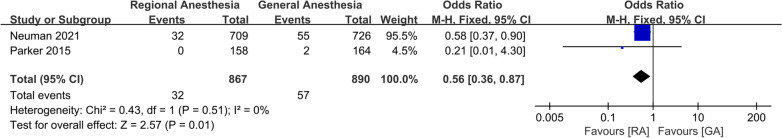
Forest plots displaying pooled effect estimates for acute myocardial infarction. *RA* Regional anesthesia, *GA* General anesthesia

**Fig. 11:**
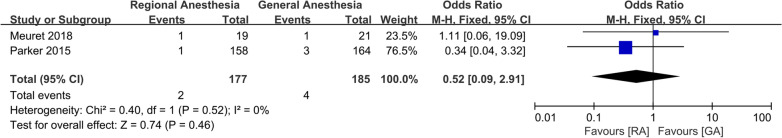
Forest plots displaying pooled effect estimates for pneumonia. *RA* Regional anesthesia, *GA* General anesthesia

**Fig. 12:**
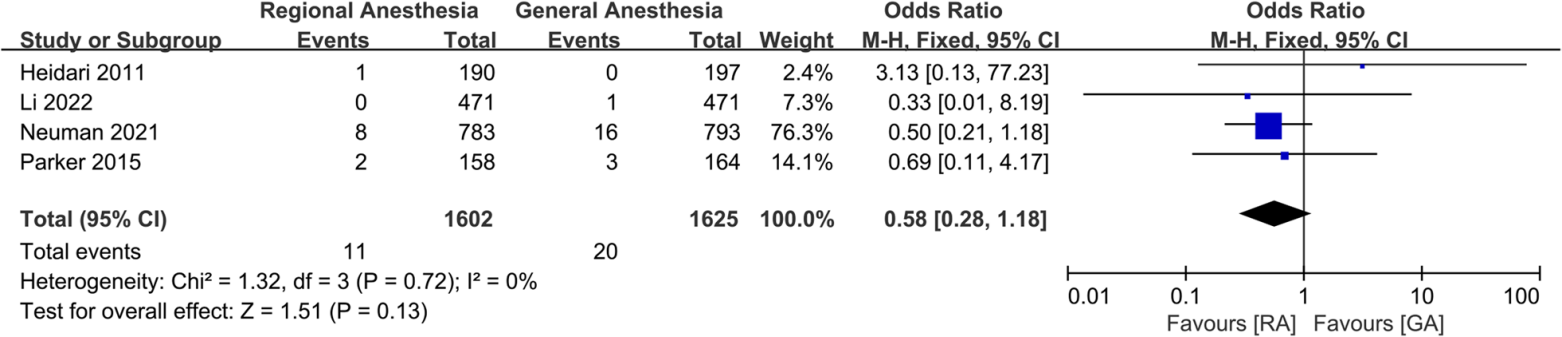
Forest plots displaying pooled effect estimates for deep vein thrombosis. *RA* Regional anesthesia, *GA* General anesthesia

**Fig. 13:**
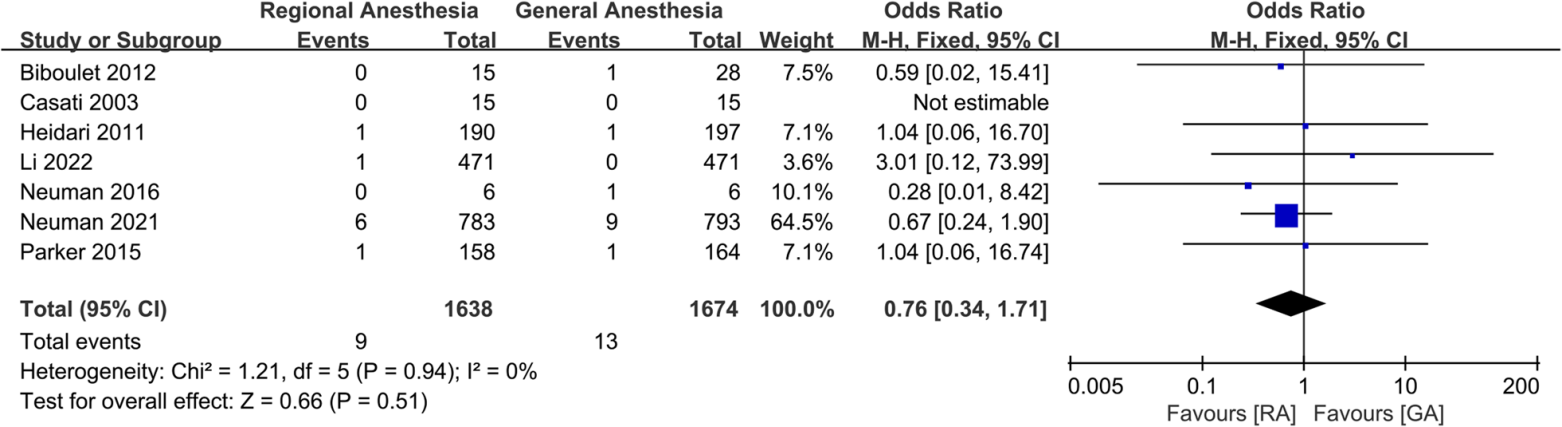
Forest plots displaying pooled effect estimates for acute kidney injury. *RA* Regional anesthesia, *GA* General anesthesia

**Fig. 14:**
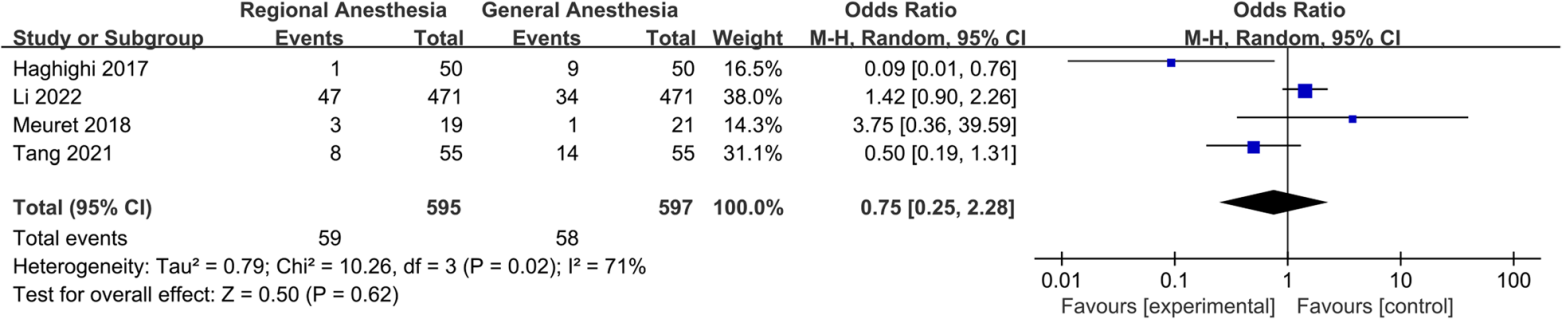
Forest plots displaying pooled effect estimates for postoperative nausea and vomiting. *RA* Regional anesthesia, *GA* General anesthesia

**Fig. 15:**
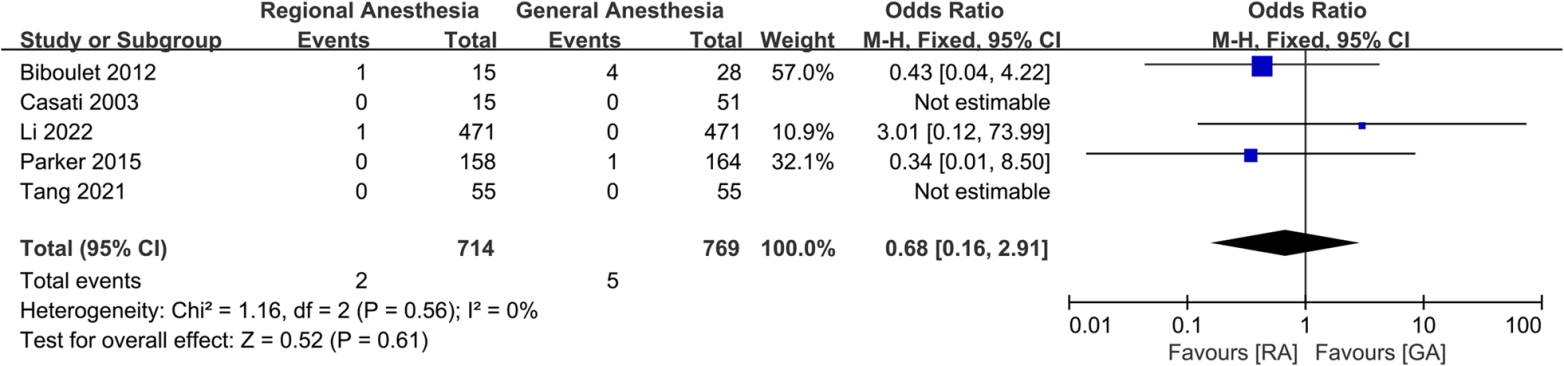
Forest plots displaying pooled effect estimates for heart failure. *RA* Regional anesthesia, *GA* General anesthesia

**Fig. 16:**
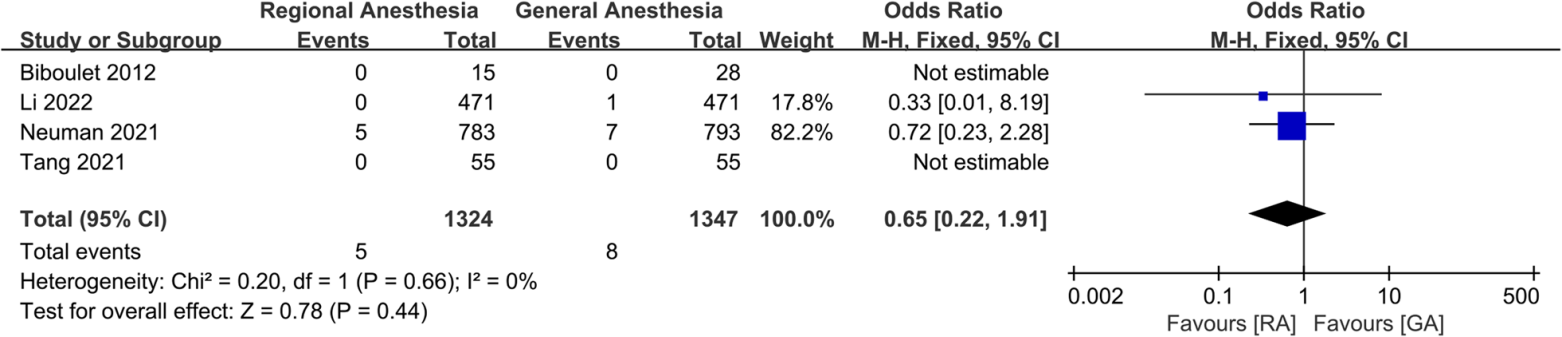
Forest plots displaying pooled effect estimates for stroke, *RA* Regional anesthesia, *GA* General anesthesia

**Fig. 17:**
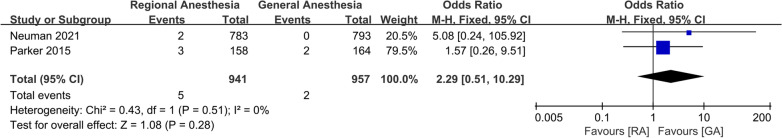
Forest plots displaying pooled effect estimates for surgical-site infection. *RA* Regional anesthesia, *GA* General anesthesia

For postoperative complications, a meta-analysis of two studies showed that there was a significant increase in the incidence of AKI in patients receiving GA (OR 0.56; 95% CI 0.36, 0.87, *P* = 0.01, *n* = 1757) (Fig. 10). There was no significant difference between the two groups in the incidence of DVT (OR 0.52; 95% CI 0.09, 2.91, *P* = 0.46, *n* = 362) (Fig. 11), pneumonia (OR 0.58; 95% CI 0.28, 1.18, *P* = 0.13, *n* = 3,227) (Fig. 12), acute myocardial infarction (OR 0.76; 95% CI 0.34, 1.71, *P* = 0.51, *n* = 3312) (Fig. 13), PONV (OR 0.75; 95% CI 0.25, 2.28, *P* = 0.62, *n* = 1192) (Fig. 14), heart failure (OR 0.68; 95% CI 0.16, 2.91, *P* = 0.61, *n* = 1483) (Fig. 15), stroke (OR 0.65; 95% CI 0.22, 1.91, *P* = 0.44, *n* = 2671) (Fig. 16), and surgical-site infection (OR 2.29; 95% CI 0.51, 10.29, *P* = 0.28, *n* = 1898) (Fig. 17). The quality of evidence was graded as moderate for acute myocardial infarction and stroke, and low for PONV, DVT, heart failure, pneumonia, surgical-site infection, and AKI (Additional file 2).

### Sensitivity analysis and subgroup analysis

Sensitivity analyses were conducted via leave-one-out analysis in terms of operative time and intraoperative blood loss. The conclusion was observed to change and the I^2^ was reduced from 71 to 39% after excluding the study of Haghighi and colleagues [25] in operative time (WMD: − 3.49; 95% CI − 7.60, − 0.63, *P* = 0.10, *n* = 2291). After excluding the other three studies, respectively [24, 28, 32], the conclusion changed, but the *I*^2^ was not less than 50%. Sensitivity analyses of blood loss did not change the overall results of the pooled analysis.

Nine studies [16, 23–25, 27–31] used SA as the sole regional anesthetic technique without sedation and were included in the subgroup analysis. The conclusion changed in LOS in the meta-analysis of two studies and demonstrated no significant difference between the two groups (WMD: − 0.10; 95% CI − 0.29, 0.08, *P* = 0.28, *n* = 422). No significant difference was found in postoperative pain scores in the subgroup analysis of the two studies (WMD: − 1.59; 95% CI − 3.38, 0.21, *P* = 0.08, *n* = 210). The other pooled results were consistent with previous outcomes when all of the studies were considered.

### Publication bias

**Fig. 18:**
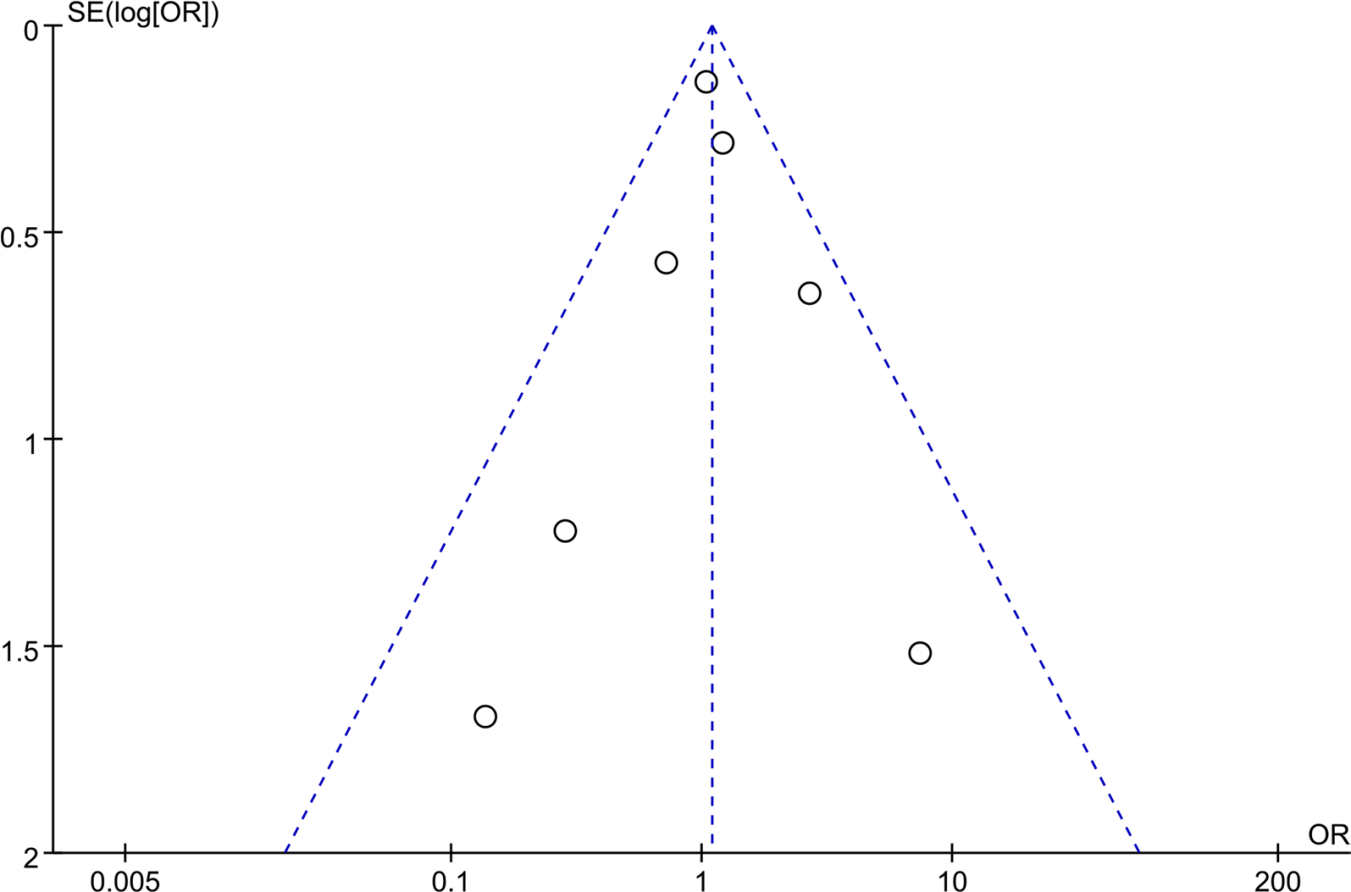
Funnel plot displaying publication bias for incidence of delirium

**Fig. 19:**
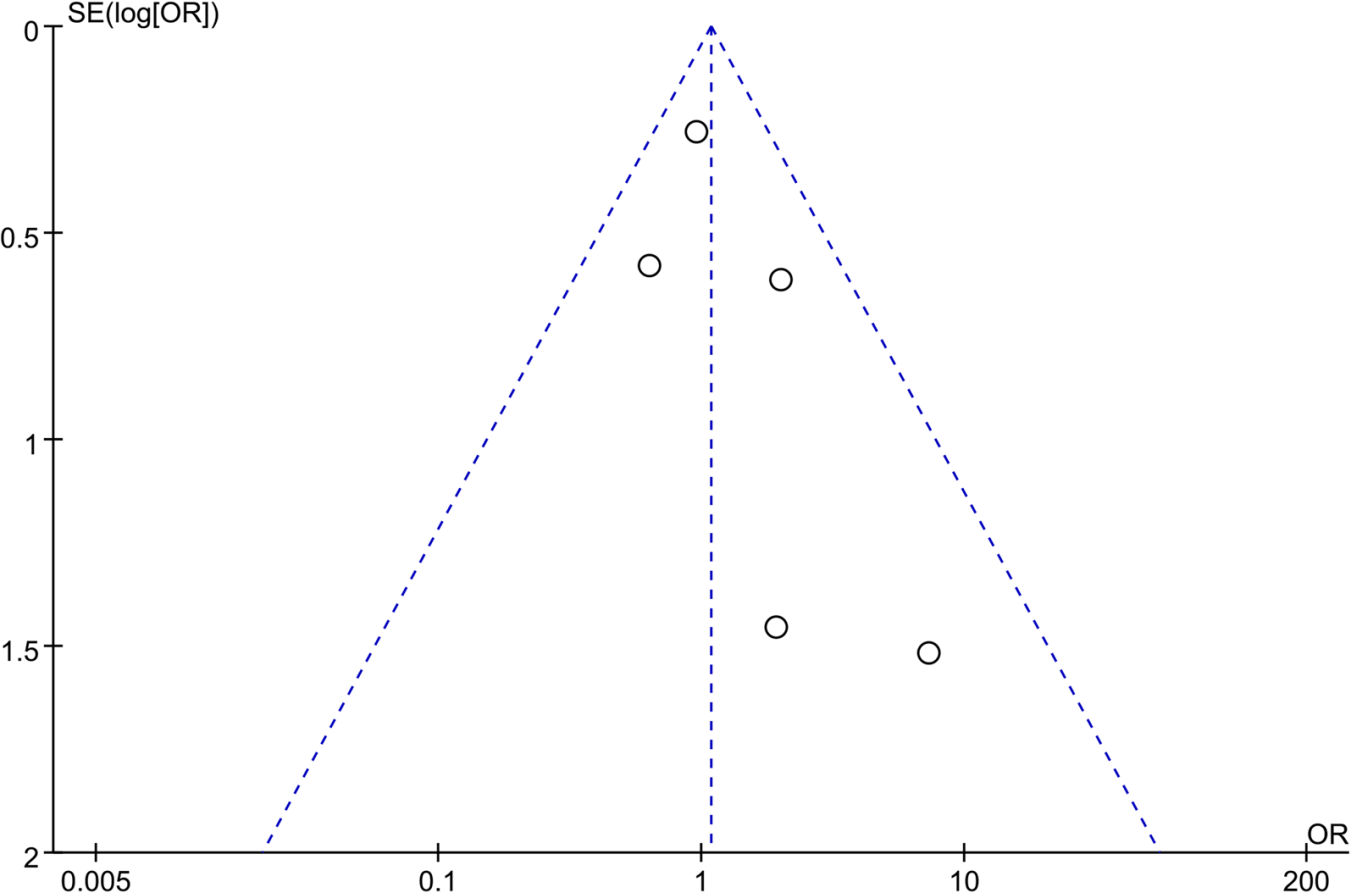
Funnel plot displaying publication bias for mortality

The funnel plot and the Egger test for the incidence of delirium (Fig. 18, *P* = 0.91) and mortality (Fig. 19, *P* = 0.19) did not show any significant publication bias.

## Discussion

This study included a total of thirteen RCTs with 3736 patients comparing the difference between RA and GA in hip fracture surgery. Compared to other previous studies [14, 33], our study included the latest RCTs [15–17], which had a larger sample size and focused on more comparable outcomes to thoroughly evaluate the effects of RA and GA in hip fracture surgery. For primary outcomes, our study did not observe a significant difference in the postoperative incidence of delirium or postoperative mortality. However, our meta-analysis statistically demonstrated that patients receiving GA in hip fracture surgery had a longer operative time, larger amount of intraoperative blood loss, higher postoperative pain scores, longer LOS, and higher incidence of acute kidney injury than those receiving RA. No significant difference was found in other perioperative outcomes and complications.

Postoperative delirium is a common complication associated with hip fracture repair that may significantly increase mortality, morbidity, functional and cognitive decline, and healthcare costs [34–36]. In our study, a meta-analysis of seven RCTs demonstrated no significant difference in postoperative delirium for older patients undergoing hip fracture surgery, and the conclusion did not change in the pooled results of the CAM group. This result was consistent with previous RCTs [15–17] and meta-analyses [14, 33]. As sedation affected the incidence of postoperative delirium, we also excluded two studies [17, 26] with sedation but did not observe any change in the conclusion. The criteria in the DSM-V or the WHO ICD-10 classification of diseases were the standard practice for the diagnosis of delirium; however, it was difficult to conduct these classifications in clinical situations [37]. CAM and other tools are commonly used in clinical trials to screen for delirium [38]. Postoperative delirium is related to many factors, such as age, ASA physical status, preexisting diseases, surgery, anesthesia, and other risk factors [34, 39]. Moreover, studies that adjusted for confounders had difficulty identifying all of the risk factors. Therefore, the potential benefits of certain types of anesthesia for postoperative delirium remain uncertain.

Regarding postoperative mortality, previous studies have failed to reach a consensus on the mortality benefit of RA and GA in patients undergoing hip fracture surgery. Mcisaac and colleagues indicated that RA increased survival rates at 30 days in patients undergoing hip fracture surgery in hospitals with more than 20–25% RA use [40]. An overview of Cochrane systematic reviews showed that RA was related to reduced 30-day mortality [41]. However, there was no significant difference between the two groups in two recent meta-analyses [14, 33]. In addition, in our study, we did not observe any difference in postoperative mortality or 30-day mortality. The in-hospital mortality was not analyzed due to a lack of comparable data. Van and colleagues [42] demonstrated a significantly higher incidence of in-hospital mortality but no significant difference in 30-day mortality in the GA group which indicated that in-hospital mortality may be more sensitive to the effect of anesthetic techniques. However, the conclusion was limited because they included one RCT and four observational studies; in addition, one study [43] included considerably more patients than others which was weighted in the analysis at 53.2%. More RCTs comparing the effects of RA with GA on in-hospital mortality and postoperative mortality are needed to verify the conclusion.

In this study, we demonstrated that the RA group was associated with less blood loss, which was consistent with the results of previous studies [14, 44]. In addition, the result seemed to be robust according to the sensitivity analysis. The reason for this effect may be related to hemodynamic changes. RA can lead to a reduction in blood pressure and heart rates [32, 45]; in addition, for patients with GA, there may be an increase in venous blood pressure [46]. The shorter operative time could also lead to less blood loss in the RA group. However, the operative time results in this study was not robust and need to be interpreted with caution. The reduced intraoperative blood loss may cause a decreased incidence of blood transfusion. However, in this study, blood transfusion was not significantly different between the two groups. The trigger of blood transfusion is still debated [47]. Many studies have not reported blood transfusion as an outcome; moreover, in the five studies that reported on blood transfusion, only one study reported the trigger of blood transfusion [24]. Therefore, the evidence on blood transfusion was inconclusive.

Intraoperative hypotension can cause hypoperfusion and organ damage. Several studies have indicated that the incidence of intraoperative hypotension in the GA group was higher than that in the RA group in hip fracture surgery [24, 48]. The study conducted by Li and colleagues had the largest sample size of RCTs reporting on intraoperative hypotension to date, in which they found that the incidence of intraoperative hypotension was significantly higher in the GA group than in the RA group [15]. However, this result was in contrast with the findings in our study. Our findings were graded as low according to the GRADE system, and the heterogeneity was high. Moreover, the definition of intraoperative hypotension varied among studies; thus, so it was difficult to conduct a meta-analysis with high-quality evidence.

It is known that acute postoperative pain following orthopedic surgery is more severe in patients receiving GA than in those receiving RA [49, 50]. Two studies [25, 32] specifically demonstrated significantly lower postoperative pain scores in the RA group for patients undergoing hip fracture surgery. In addition, a previous systematic review [51] revealed a consistent result. However, this review was limited to the inclusion of studies conducted before 2000 [51]. Li and colleagues assessed pain scores with VAS ranging from 0 to 100 and did not observe a significant difference between the two groups [15]. Postoperative pain scores assessed with VAS ranging from 0 to 10 were included in the meta-analysis and demonstrated significantly lower pain scores in patients receiving RA than in those receiving GA. However, due to the small sample size and high heterogeneity in our analysis, the conclusion should be interpreted with caution.

It has been reported that a decrease in postoperative pain severity was associated with a shorter LOS [52]. LOS is one of the most widely reported outcomes. Several observational studies have indicated a shorter LOS in patients receiving RA [40, 53, 54]. However, the effect of anesthesia techniques on the LOS was observed to be controversial in systematic reviews [14, 33, 42, 55]. In our analysis of RCTs, we did not detect any significant difference in the LOS between the two groups. The results of a meta-analysis of LOS can be affected by different definitions and healthcare systems; in addition, the fracture type and surgical procedures are also important factors [33, 56].

Kim and colleagues found that RA was associated with a lower risk of AKI in patients undergoing TKA [57]. In our study, for older patients undergoing hip fracture surgery, the meta-analysis of two studies showed a significantly lower incidence of AKI in the RA group. Due to the fact that only two studies were included, as well as the fact that the regional versus general anesthesia for promoting independence after hip fracture (REGAIN) study conducted by Newman and colleagues was weighted in the analysis at 95.5%, the conclusion was limited. A systematic review demonstrated that the incidence of acute myocardial infarction was lower under RA than under GA, which was observed in the analysis including observational studies [42]. However, in our analysis of RCTs involving a total of 3312 patients, no significant difference was found between the two groups, and the quality of the evidence was moderate. Haghighi and colleagues demonstrated that PONV was significantly lower in the RA group than in the GA group [25]. The reason for this result may be related to better postoperative analgesia and the decrease in opioid consumption in the RA group [58]. In our study, no difference in PONV for older patients undergoing hip fracture surgery was detected. However, the heterogeneity was high and PONV was graded as low in the GRADE system.

This systematic review and meta-analysis had several potential limitations. First, seven of thirteen included studies were small studies with a sample size of < 100 patients. Some results of meta-analyses should be interpreted with caution due to the small-study effects [59]. Second, almost all of the RCTs were open-label RCTs, and the influence on our results cannot be excluded. Third, the types and dosages of the utilized anesthetics used varied across the studies, which cannot be controlled for. Fourth, several definitions of outcomes were inconsistent, such as intraoperative hypotension and LOS, which may be the source of the high heterogeneity. In addition, the study only searched studies in the English language and may have missed potentially relevant studies in the non-English literature.

## Conclusion

In our study, RA did not significantly reduce the incidence of postoperative delirium or mortality in older patients undergoing hip fracture surgery compared to GA. Due to the limitations of this study, the evidence on delirium and mortality is still inconclusive. Nevertheless, we found that RA could reduce the operative time, amount of intraoperative blood loss, postoperative pain score, LOS, and risk of AKI. Although the results showed statistically significant differences in operative time and blood loss, we consider it may not have clinical significance.

## Supplementary Information

**Additional file 1**. Risk of bias for randomized studies assessed by the Cochrane Collaboration risk of bias tool.

**Additional file 2**. The GRADE evidence quality for all outcomes.

## Data Availability

All data generated or analyzed during this study are included in this published article and its supplementary information files.

## Abbreviations

GA: General anesthesia
LOS: Length of stay
RA: Regional anesthesia
RCTs: Randomized controlled trials
GRADE: Grading of Recommendations, Assessment, Development, and Evaluations
PRISMA: Preferred Reporting Items for Systematic Reviews and Meta-Analyses
SA: Spinal anesthesia
PONV: Postoperative nausea and vomiting
DVT: Deep vein thrombosis
AKI: Acute kidney injury
ASA: American Society of Anesthesiologists
WMDs: Weighted mean differences
SDs: Standard deviations
ORs: Odds ratios
CIs: Confidence intervals
IQR: Interquartile range
CAM: Confusion Assessment Method
MMSE: Mini Mental State Examination test
VAS: Visual analog scale
