# Supplementary material for: Regional versus general anesthesia in older patients for hip fracture surgery: a systematic review and meta-analysis of randomized controlled trials
Source: J Orthop Surg Res. 2023 Jun 13;18:428. doi: 10.1186/s13018-023-03903-5 (PMC10262548; doi:10.1186/s13018-023-03903-5)
Supplement: Supplementary file 1 — Additional file 1. Risk of bias for randomized studies assessed by the Cochrane Collaboration risk of bias tool. [file 13018_2023_3903_MOESM1_ESM.docx]

**Additional file 1:** Risk of bias for randomized studies assessed by the Cochrane Collaboration risk of bias tool.

**
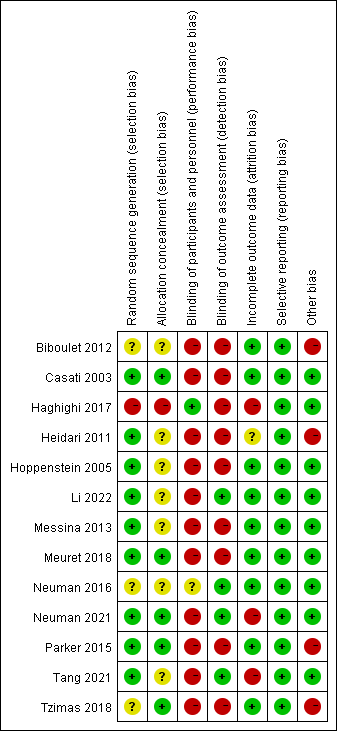
**

**Li 2022**

| Risk of bias | Author’s judgement | Support for judgement |
| --- | --- | --- |
| Random sequence generation (selection bias) | Low risk | Randomization process explained |
| Allocation concealment (selection bias) | Unclear risk | No information |
| Blinding of participants and personnel (performance bias) | High risk | Study was open label |
| Blinding of outcome assessment (detection bias) | Low risk | Outcome assessors blinded. |
| Incomplete outcome data (attrition bias) | Low risk | All missing data considered |
| Selective reporting (reporting bias) | Low risk | All results presented as per method |
| Other bias | Low risk | Groups well balanced |

**Neuman 2021**

| Risk of bias | Author’s judgement | Support for judgement |
| --- | --- | --- |
| Random sequence generation (selection bias) | Low risk | Randomization process explained |
| Allocation concealment (selection bias) | Low risk | randomization assignment from the data-management system |
| Blinding of participants and personnel (performance bias) | High risk | No information |
| Blinding of outcome assessment (detection bias) | Low risk | Outcome assessors blinded. |
| Incomplete outcome data (attrition bias) | High risk | The missing data was considerable. |
| Selective reporting (reporting bias) | Low risk | All results presented as per method |
| Other bias | Low risk | Groups well balanced |

**Tang 2021**

| Risk of bias | Author’s judgement | Support for judgement |
| --- | --- | --- |
| Random sequence generation (selection bias) | Low risk | Randomization process explained |
| Allocation concealment (selection bias) | Unclear risk | No information |
| Blinding of participants and personnel (performance bias) | High risk | No information |
| Blinding of outcome assessment (detection bias) | Low risk | Outcome assessors blinded |
| Incomplete outcome data (attrition bias) | High risk | All data considered |
| Selective reporting (reporting bias) | Low risk | All results presented as per method |
| Other bias | Low risk | Groups well balanced |

**Meuret 2018**

| Risk of bias | Author’s judgement | Support for judgement |
| --- | --- | --- |
| Random sequence generation (selection bias) | Low risk | Randomization process explained |
| Allocation concealment (selection bias) | Low risk | Sealed envelopes |
| Blinding of participants and personnel (performance bias) | High risk | Study was open label |
| Blinding of outcome assessment (detection bias) | High risk | No information |
| Incomplete outcome data (attrition bias) | Low risk | No patient lost to follow up |
| Selective reporting (reporting bias) | Low risk | All results presented as per method |
| Other bias | Low risk | Groups well balanced |

**Tzimas 2018**

| Risk of bias | Author’s judgement | Support for judgement |
| --- | --- | --- |
| Random sequence generation (selection bias) | Unclear risk | No information |
| Allocation concealment (selection bias) | Low risk | Closed envelopes |
| Blinding of participants and personnel (performance bias) | High risk | No information |
| Blinding of outcome assessment (detection bias) | High risk | No information |
| Incomplete outcome data (attrition bias) | Low risk | A small amount of patient lost to follow up |
| Selective reporting (reporting bias) | Low risk | All results presented as per method |
| Other bias | High risk | Study not on intention-to-treat basis |

**Haghighi 2017**

| Risk of bias | Author’s judgement | Support for judgement |
| --- | --- | --- |
| Random sequence generation (selection bias) | High risk | Randomization using sequential numbers |
| Allocation concealment (selection bias) | High risk | Sequential numbers |
| Blinding of participants and personnel (performance bias) | Low risk | Double blind |
| Blinding of outcome assessment (detection bias) | High risk | No information |
| Incomplete outcome data (attrition bias) | High risk | No information |
| Selective reporting (reporting bias) | Low risk | All results presented as per method |
| Other bias | Low risk | Groups well balanced |

**Neuman 2016**

| Risk of bias | Author’s judgement | Support for judgement |
| --- | --- | --- |
| Random sequence generation (selection bias) | Unclear risk | No information |
| Allocation concealment (selection bias) | Unclear risk | No information |
| Blinding of participants and personnel (performance bias) | Unclear risk | No information |
| Blinding of outcome assessment (detection bias) | Low risk | Outcome assessors blinded |
| Incomplete outcome data (attrition bias) | Low risk | No patient lost to follow up |
| Selective reporting (reporting bias) | Low risk | All results presented as per method |
| Other bias | Low risk | Groups well balanced |

**Casati 2003**

| Risk of bias | Author’s judgement | Support for judgement |
| --- | --- | --- |
| Random sequence generation (selection bias) | Low risk | Randomization mentioned; detail minimal |
| Allocation concealment (selection bias) | Low risk | Sealed envelopes |
| Blinding of participants and personnel (performance bias) | High risk | No information |
| Blinding of outcome assessment (detection bias) | High risk | No information |
| Incomplete outcome data (attrition bias) | Low risk | All missing data considered; no loss to follow-up |
| Selective reporting (reporting bias) | Low risk | All results presented as per method |
| Other bias | Low risk | Groups well balanced |

**Biboulet 2012**

| Risk of bias | Author’s judgement | Support for judgement |
| --- | --- | --- |
| Random sequence generation (selection bias) | Unclear risk | No details of randomization process |
| Allocation concealment (selection bias) | Unclear risk | No information |
| Blinding of participants and personnel (performance bias) | High risk | No information |
| Blinding of outcome assessment (detection bias) | High risk | No information |
| Incomplete outcome data (attrition bias) | Low risk | All missing data considered |
| Selective reporting (reporting bias) | Low risk | All results presented as per method |
| Other bias | High risk | Study not on intention-to-treat basis |

**Parker 2015**

| Risk of bias | Author’s judgement | Support for judgement |
| --- | --- | --- |
| Random sequence generation (selection bias) | Low risk | Randomization process explained |
| Allocation concealment (selection bias) | Low risk | Sealed envelope |
| Blinding of participants and personnel (performance bias) | High Risk | No blinding of patient or participant |
| Blinding of outcome assessment (detection bias) | High risk | No outcome assessor blinding |
| Incomplete outcome data (attrition bias) | Low risk | All data considered; only 1 patient lost to follow up |
| Selective reporting (reporting bias) | Low risk | All outcomes reported as per method |
| Other bias | High risk | GA group had more male patients |

**Heidari 2011**

| Risk of bias | Author’s judgement | Support for judgement |
| --- | --- | --- |
| Random sequence generation (selection bias) | Low risk | Randomization process explained |
| Allocation concealment (selection bias) | Unclear risk | No information |
| Blinding of participants and personnel (performance bias) | High risk | No information |
| Blinding of outcome assessment (detection bias) | High risk | No information |
| Incomplete outcome data (attrition bias) | Unclear risk | All missing data considered |
| Selective reporting (reporting bias) | Low risk | All results presented as per method |
| Other bias | High risk | Not based on intention-to-treat principle: patients excluded because of change in anaesthetic/ surgical plan |

**Messina 2013**

| Risk of bias | Author’s judgement | Support for judgement |
| --- | --- | --- |
| Random sequence generation (selection bias) | Low risk | Randomization process explained |
| Allocation concealment (selection bias) | Unclear risk | No information |
| Blinding of participants and personnel (performance bias) | High risk | No information |
| Blinding of outcome assessment (detection bias) | High risk | No information |
| Incomplete outcome data (attrition bias) | Low risk | All data considered; no loss to follow-up |
| Selective reporting (reporting bias) | Low risk | All results presented as per method with no deviation from intended intervention |
| Other bias | Low risk | Groups well balanced |

**Hoppenstein 2005**

| Risk of bias | Author’s judgement | Support for judgement |
| --- | --- | --- |
| Random sequence generation (selection bias) | Low risk | Randomization process explained |
| Allocation concealment (selection bias) | Unclear risk | No details on allocation concealment |
| Blinding of participants and personnel (performance bias) | High risk | Study was open label |
| Blinding of outcome assessment (detection bias) | High risk | Study was open label |
| Incomplete outcome data (attrition bias) | Low risk | All data considered |
| Selective reporting (reporting bias) | Low risk | All results presented as per method |
| Other bias | Low risk | Groups well balanced |
