# Supplementary material for: Regional versus general anesthesia in older patients for hip fracture surgery: a systematic review and meta-analysis of randomized controlled trials
Source: J Orthop Surg Res. 2023 Jun 13;18:428. doi: 10.1186/s13018-023-03903-5 (PMC10262548; doi:10.1186/s13018-023-03903-5)
Supplement: Supplementary file 2 — Additional file 2. The GRADE evidence quality for all outcomes. [file 13018_2023_3903_MOESM2_ESM.docx]

**Additional file 2:** The GRADE evidence quality for all outcomes

| **Certainty assessment** | | | | | | | **№ of patients** | | **Effect** | | **Certainty** | **Importance** |
| --- | --- | --- | --- | --- | --- | --- | --- | --- | --- | --- | --- | --- |
| **№ of studies** | **Study design** | **Risk of bias** | **Inconsistency** | **Indirectness** | **Imprecision** | **Other considerations** | **RA** | **GA** | **Relative (95% CI)** | **Absolute (95% CI)** |  |  |
| **incidence of delirium** | | | | | | | | | | | | |
| 6 | randomized trials | serious | not serious | not serious | serious | none | 178/1360 (13.1%) | 162/1357 (11.9%) | **OR 1.11** (0.88 to 1.40) | **11 more per 1,000** (from 13 fewer to 40 more) | ⨁⨁◯◯ Low | IMPORTANT |
| **CAM-delirium** | | | | | | | | | | | | |
| 4 | randomized trials | not serious | not serious | not serious | not serious | none | 45/569 (7.9%) | 38/564 (6.7%) | **OR 1.18** (0.75 to 1.84) | **11 more per 1,000** (from 16 fewer to 50 more) | ⨁⨁⨁⨁ High | CRITICAL |
| **operative time** | | | | | | | | | | | | |
| 9 | randomized trials | serious | serious | not serious | serious | none | 1187 | 1204 | - | MD **4.74 lower** (8.85 lower to 0.63 lower) | ⨁◯◯◯ Very low | IMPORTANT |
| **intraoperative hypotension** | | | | | | | | | | | | |
| 5 | randomized trials | serious | not serious | not serious | serious | none | 197/718 (27.4%) | 429/726 (59.1%) | **OR 0.36** (0.11 to 1.24) | **249 fewer per 1,000** (from 454 fewer to 51 more) | ⨁⨁◯◯ Low | IMPORTANT |
| **duration of anesthesia** | | | | | | | | | | | | |
| 6 | randomized trials | serious | not serious | not serious | serious | none | 1513 | 1544 | - | MD **0.75 lower** (3.09 lower to 1.58 higher) | ⨁⨁◯◯ Low | IMPORTANT |
| **Blood loss** | | | | | | | | | | | | |
| 6 | randomized trials | serious | serious | not serious | not serious | none | 1048 | 1059 | - | MD **76.65 lower** (130.3 lower to 23 lower) | ⨁⨁◯◯ Low | NOT IMPORTANT |
| **intraoperative blood transfusion** | | | | | | | | | | | | |
| 5 | randomized trials | serious | not serious | not serious | not serious | none | 143/740 (19.3%) | 144/744 (19.4%) | **OR 0.97** (0.73 to 1.28) | **5 fewer per 1,000** (from 44 fewer to 41 more) | ⨁⨁⨁◯ Moderate | IMPORTANT |
| **mortality** | | | | | | | | | | | | |
| 6 | randomized trials | serious | not serious | not serious | serious | none | 47/1606 (2.9%) | 45/1643 (2.7%) | **OR 1.08** (0.71 to 1.64) | **2 more per 1,000** (from 8 fewer to 17 more) | ⨁⨁◯◯ Low | IMPORTANT |
| **30-day mortality** | | | | | | | | | | | | |
| 3 | randomized trials | serious | not serious | not serious | not serious | none | 14/642 (2.2%) | 13/656 (2.0%) | **OR 1.15** (0.53 to 2.47) | **3 more per 1,000** (from 9 fewer to 28 more) | ⨁⨁⨁◯ Moderate | CRITICAL |
| **postoperative pain score** | | | | | | | | | | | | |
| 3 | randomized trials | serious | not serious | not serious | not serious | none | 295 | 302 | - | MD **1.77 lower** (2.79 lower to 0.74 lower) | ⨁⨁⨁◯ Moderate | IMPORTANT |
| **length of hospitalization** | | | | | | | | | | | | |
| 4 | randomized trials | serious | serious | not serious | serious | none | 681 | 683 | - | MD **0.89 lower** (2.41 lower to 0.64 higher) | ⨁◯◯◯ Very low | NOT IMPORTANT |
| **PONV (postoperative Nausea and vomiting)** | | | | | | | | | | | | |
| 4 | randomized trials | serious | not serious | not serious | serious | none | 59/595 (9.9%) | 58/597 (9.7%) | **OR 0.75** (0.25 to 2.28) | **22 fewer per 1,000** (from 71 fewer to 100 more) | ⨁⨁◯◯ Low | IMPORTANT |
| **Acute Myocardial infarction** | | | | | | | | | | | | |
| 7 | randomized trials | serious | not serious | not serious | not serious | none | 9/1638 (0.5%) | 13/1674 (0.8%) | **OR 0.76** (0.34 to 1.71) | **2 fewer per 1,000** (from 5 fewer to 5 more) | ⨁⨁⨁◯ Moderate | CRITICAL |
| **Stroke** | | | | | | | | | | | | |
| 4 | randomized trials | serious | not serious | not serious | not serious | none | 5/1324 (0.4%) | 8/1347 (0.6%) | **OR 0.65** (0.22 to 1.91) | **2 fewer per 1,000** (from 5 fewer to 5 more) | ⨁⨁⨁◯ Moderate | IMPORTANT |
| **heart failure** | | | | | | | | | | | | |
| 5 | randomized trials | serious | not serious | not serious | serious | none | 2/714 (0.3%) | 5/769 (0.7%) | **OR 0.68** (0.16 to 2.91) | **2 fewer per 1,000** (from 5 fewer to 12 more) | ⨁⨁◯◯ Low | IMPORTANT |
| **pneumonia** | | | | | | | | | | | | |
| 4 | randomized trials | serious | not serious | not serious | serious | none | 11/1602 (0.7%) | 20/1625 (1.2%) | **OR 0.58** (0.28 to 1.18) | **5 fewer per 1,000** (from 9 fewer to 2 more) | ⨁⨁◯◯ Low | NOT IMPORTANT |
| **deep vein thrombosis** | | | | | | | | | | | | |
| 2 | randomized trials | serious | not serious | not serious | not serious | none | 2/177 (1.1%) | 4/185 (2.2%) | **OR 0.52** (0.09 to 2.91) | **10 fewer per 1,000** (from 20 fewer to 39 more) | ⨁⨁⨁◯ Moderate | IMPORTANT |
| **Surgical-site infection** | | | | | | | | | | | | |
| 2 | randomized trials | serious | not serious | not serious | serious | none | 5/941 (0.5%) | 2/957 (0.2%) | **OR 2.29** (0.51 to 10.29) | **3 more per 1,000** (from 1 fewer to 19 more) | ⨁⨁◯◯ Low | IMPORTANT |
| **Acute kidney injury** | | | | | | | | | | | | |
| 2 | randomized trials | serious | not serious | not serious | serious | none | 32/867 (3.7%) | 57/890 (6.4%) | **OR 0.56** (0.36 to 0.87) | **27 fewer per 1,000** (from 40 fewer to 8 fewer) | ⨁⨁◯◯ Low | NOT IMPORTANT |

**RA:** regional anesthesia; **GA:** general anesthesia; **CI:** confidence interval; **MD:** mean difference; **OR:** odds ratio
